# Advances in Food Waste Biomass Transformation into High-Value Products

**DOI:** 10.3390/foods13091393

**Published:** 2024-05-01

**Authors:** Umile Gianfranco Spizzirri, Donatella Restuccia

**Affiliations:** 1 Ionian Department of Law, Economics and Environment, University of Bari Aldo Moro, 74123 Taranto, Italy; g.spizzirri@unical.it; 2 Department of Management, Sapienza University of Rome, Via del Castro Laurenziano 9, 00161 Rome, Italy

## 1. Introduction

In recent years, there has been a concerning surge in waste generation, with agri-food waste emerging as a significant issue across various stages of the food supply chain [1]. This waste, arising from production, processing, distribution, and consumption, not only leads to substantial economic losses but also poses ethical and environmental challenges [2]. Addressing this modern challenge requires minimizing waste generation, optimizing production processes, and transitioning to a circular economic model, emphasizing minimal waste generation and the “zero waste concept” [3,4]. One promising approach involves valorizing agri-food waste and byproducts to extract valuable compounds for nutraceutical, agricultural, and food applications [5,6,7,8]. Additionally, there is a focus on developing new formulations of food products with enhanced nutritional, technological, and sensory qualities [9,10]. Moreover, economic evaluations and assessments of consumer acceptance play crucial roles in gauging the viability and marketability of high-value products derived from agri-food waste and byproducts [11,12]. This Special Issue aims to delve into these areas, exploring innovative strategies to harness the potential of agri-food waste for sustainable and value-added solutions in the food industry.

## 2. An Overview of the Published Articles

This Special Issue explores the potential of various waste materials from the food industry and agriculture to yield valuable compounds, thereby contributing to sustainable practices and environmental conservation. It encompasses several studies and reviews, each focusing on different aspects of waste valorization and circular economy models. Food waste represents a valuable source of active molecules such as polyphenols and anthocyanins useful in the preparation of high-value industrial products with remarkable health proprieties.

The extraction of bioactive compounds from red and blonde orange peels as well as lemon peels using hydroalcoholic solutions with ultrasound assistance was investigated. The extracts were used for the formulation of functional gummies that demonstrated significant antioxidant properties. The study highlighted the efficient utilization of highly polluting waste materials as valuable sources of bioactive compounds for value-added food preparation [contribution 1]. Another research work investigated defatted rosehip seed waste as a source of polyphenol molecules. In this case, supercritical carbon dioxide and natural deep eutectic solvent extractions were employed to recover phenolic compounds from the seed waste. The environmentally friendly processes transformed the waste materials into functional products with potential applications in the food industry, also contributing to sustainable production practices [contribution 2].

Anthocyanins are natural pigments found in various fruits and vegetables, providing red, violet, and blue hues. They serve as healthier alternatives to artificial food colorants, potentially offering antioxidant and other health benefits, such as anticarcinogenic and cardioprotective effects. Additionally, anthocyanins can extend the shelf life of processed foods while enhancing their nutritional value. Chokeberry pomace, rich in anthocyanins, underwent enzyme-assisted extraction to produce natural food colorants with antioxidant properties. The study demonstrated the viability of recovering and purifying anthocyanins from pomace, presenting a sustainable valorization strategy for byproducts from the juice industry [contribution 3]. Moreover, eggplant peel waste was investigated for its high anthocyanin content, optimizing solvent compositions to yield extracts rich in antioxidants and pigments. This study demonstrated the potential of utilizing agricultural waste for the extraction of beneficial compounds, contributing to waste reduction and sustainable production practices [contribution 4].

Additionally, waste melons were utilized in the production of fruit-based wines, showcasing a circular economy model in the food industry. The resulting wines exhibited enhanced aromatic profiles and satisfactory sensory evaluation, presenting a novel approach to transforming rejected fresh fruits into high-value products [contribution 5].

Furthermore, waste walnut sawdust was evaluated as a substrate for cultivating Auricularia auricula mushrooms, demonstrating improved growth and nutritional quality compared to traditional substrates. This study emphasized the potential of utilizing agricultural waste for mushroom cultivation, providing a new avenue for waste valorization [contribution 6].

Increased global interest in soursop (*Annona muricata*) underscores its byproducts’ potential, rich in phytochemicals, for industrial use, aiding environmental sustainability and income generation. These byproducts, including damaged fruits, seeds, peels, and leaves, often discarded, hold promise for valorization into products like soursop powder, bioactive compounds, and biochar [contribution 7]. Green transition and circular economy principles were emphasized, utilizing brewer’s spent grain, coffee grounds, burdock, and willow for valuable compounds’ extraction, promoting sustainable biowaste utilization with cutting-edge green technologies [contribution 8].

Two reviews completed the Special Issue. Firstly, the production of xylooligosaccharides (XOS) from lignocellulosic biomass was examined, focusing on its prebiotic and therapeutic properties. The integration of XOS manufacturing into biorefineries was evaluated from an economic and environmental sustainability point of view [contribution 9]. Lastly, a review discussed the innovative applications of 3D-printing technology for the valorization of agri-food processing waste, underlining its potential to obtain edible foods and biodegradable materials for packaging and related uses [contribution 10].

Overall, these studies and reviews collectively underscored the importance of adopting sustainable practices in the food industry and exploiting waste materials to create value-added products. Through innovative approaches and technological advancements, the potential for waste valorization and circular economy models in the food industry can be fully implemented, leading to a more sustainable and resource-efficient future.
